# Comparison of the Anaesthesia Success Rate in Maxillary First and Second Molars with 3% Prilocaine as the Anaesthetic Agent

**DOI:** 10.14744/eej.2021.74755

**Published:** 2021-12-03

**Authors:** Masoud PARIROKH, Iman SAMADI, Nouzar NAKHAEE, Paul ABBOTT

**Affiliations:** 1. Department of Endodontics, Endodontology Research Center, Kerman University of Medical Sciences, Kerman, Iran; 2. Department of Endodontics, Oral and Dental Diseases Research Center, Kerman University of Medical Sciences, Kerman, Iran; 3. Department of Social Medicine, Neuroscience Research Center, Kerman University of Medical Sciences, Kerman, Iran; 4. Department of Oral Restorative and Rehabilitative Sciences, Faculty of Dentistry, University of Western Australia, Perth, Australia

**Keywords:** Anesthesia, infiltration, irreversible pulpitis, maxillary molars, prilocaine

## Abstract

**Objective:**

The aim of this study was to compare the success rate of anaesthesia with 3% prilocaine and felypressin (0.03 IU/mL) in maxillary first and second molar teeth with irreversible pulpitis.

**Methods:**

The study population was 159 patients (53 males, 106 females) who had maxillary first or second molar teeth with irreversible pulpitis (84 first molars, 75 second molars). A buccal infiltration of 3% prilocaine with 0.03 IU/mL felypressin was used as the primary anaesthetic technique. In addition to using a categorised pain score, sound, eye movement and body motion were considered signs of anaesthesia efficacy. The data were analysed with independent t and Chi-square tests. Significance was set at α=0.05.

**Results:**

Overall, the success rate was 56.6% in maxillary molars, 53.6% in maxillary first molars, and 60% in maxillary second molars. There was no statistically significant difference between maxillary first and second molars in terms of anaesthesia success rate (P>0.05). The overall success rate of intraligament supplementary injections was 50%, and intrapulpal supplementary injections was 97.91%. No significant difference was found between maxillary first and second molars in terms of the success rate of the supplemental techniques (P>0.05).

**Conclusion:**

No significant difference was found between maxillary first and second molars in terms of anaesthesia success rate when 3% prilocaine with 0.03 IU/mL felypressin was used as an anaesthetic solution for the infiltration injection.

## Introduction

Effective pain management can be a challenge in endodontic practice (1). Therefore, numerous investigations have evaluated different anaesthetic solutions and techniques to introduce practical strategies for pain management during endodontic treatment (2-7).

Lidocaine has been the most popular anaesthetic agent employed by dentists around the world (8). However, a recent network systematic review and meta-analysis reported that lidocaine was weaker than prilocaine and articaine in terms of the success rate of anaesthesia for inferior alveolar nerve block (IANB) injections in patients with irreversible pulpitis (7). Furthermore, another study described that prilocaine has a shorter onset of anaesthesia than lidocaine in a buccal infiltration technique and can be used when lidocaine is contraindicated (9). Therefore, based on recent studies, it would be helpful to investigate prilocaine in combination with anaesthetic solutions for anaesthetising teeth with irreversible pulpitis.

It has been generally accepted that mandibular molars are the most difficult teeth to anaesthetise, especially if irreversible pulpitis is present (1, 8). Therefore, most investigations in endodontics have focused on the success of IANB injections. Several studies have compared the anaesthesia success rate in maxillary molars with irreversible pulpitis (10-15), but these studies have only evaluated maxillary first molars. However, due to the anatomical variations, such as different cortical plate thicknesses over the maxillary first and second molars and the position of the malar bone, it may be possible to have different success rates when infiltration injections are used as the primary anaesthetic technique for maxillary first and second molars (16-19).

Supplementary injections should be used when a primary anaesthesia technique has not provided adequate anaesthesia (1). However, it has been reported that the success rate of supplementary techniques is not the same in different regions of the oral cavity (20).

Therefore, the aim of the present investigation was to compare the efficacy of 3% prilocaine with 0.03 IU/mL felypressin as the anaesthetic agent for maxillary first and second molar teeth. The primary hypothesis of this clinical investigation was that there is no significant difference between anaesthesia success rates for maxillary first and the second molars. The secondary hypothesis was that there is no significant difference between the success rates of supplementary techniques for anaesthetising maxillary first and second molars.

**Highlights**

• This study compared the success rate of anaesthesia with 3% prilocaine and felypressin (0.03 IU/mL) in maxillary first and second molar teeth with irreversible pulpitis.
• A categorised scale based on patients’ need for further anaesthesia was used to evaluate the patients’ pain and anaesthesia success.
• Anaesthesia success rate between maxillary first and second molars with irreversible pulpitis was not significantly different following buccal infiltration injections of 3% prilocaine with 0.03 IU/mL felypressin.

## Materials and Methods

In a prospective investigation with Ethics Committee reference number IR.KMU.REC.1398.236, the study population were all patients with the following inclusion criteria that attended a private office limited to endodontic treatments from April 2016 to October 2019.

Sample size calculations required 70 patients in each group to detect a difference of 20% in the success rate of anaesthesia.

The inclusion criteria were males and females with ASA I and II health classification, 18 to 65 years old, maxillary first and second molars with irreversible pulpitis, and no systemic contraindication. Furthermore, they had no history of hypersensitivity to 3% prilocaine with 0.03 IU/mL felypressin, the presence of pulp bleeding after preparing the access cavity, and no history of analgesic consumption during the 12 hours before treatment. Irreversible pulpitis was defined with a history or presence of spontaneous pain and lingering pain to a cold stimulus.

The exclusion criteria were lactation or pregnancy, presence of a periapical radiolucency, sensitivity to percussion, chronic consumption of alcohol or any medication that may affect pain perception. Furthermore, unrestorable teeth, teeth with severe periodontal involvement, moderate to severe spontaneous pain, pulp necrosis, and teeth with swellings or sinus tracts were excluded.

After applying the inclusion and exclusion criteria, 159 patients (53 male, 106 female) with 84 first and 75 second maxillary molars with irreversible pulpitis were included.

Before commencing the treatment, patients were interviewed and graded with a four-category pain scale (no pain, mild pain, moderate pain, severe pain). One well-experienced practitioner performed all steps of the treatment. All patients received a complete description of the procedure and were asked to raise their hand if they felt any pain or discomfort during access cavity preparation and root canal instrumentation. In addition, during the treatment, the practitioner paid full attention to the patients’ responses, such as any sound, eye movement, or body motion (SEM) (21).

For each patient, 20% benzocaine gel (Premier, Philadelphia, PA, USA) was used as a topical agent to anaesthetise the buccal mucosa at the injection site. After 1 minute, all patients received 1.8 mL of 3% prilocaine with 0.03 IU/mL felypressin (Persocaine, Daru Pakhsh, Tehran, Iran) as the anaesthetic agent by needle penetration (27-G 25 mm needle; Nik Rahnama Kar Co, Tehran, Iran). An infiltration injection was used as the primary anaesthetic technique. The place of injection was between the mesiobuccal and the distobuccal roots of the maxillary first or second molars. The injection was slowly administered for over 1 minute. Five minutes after injection of the anaesthetic solution, the tooth was isolated with a dental dam, and the access cavity preparation was commenced. 

If a patient felt pain and raised their hand or any SEM were noted, the practitioner stopped working and asked the patient about the quality of pain and if they needed another anaesthetic injection for pain relief. The anaesthetic injection was considered a “success” if the patient had no pain or mild pain and no need for supplementary techniques. The injection was considered a “failure” if the patient had moderate to severe pain or asked for a further anaesthetic injection. If a patient’s initial response was “success” but later raised a hand or had a positive SEM response following the treatment without supplementary injection, the anaesthetic injection was categorised as a “failure”, and a supplementary injection was administered. Supplementary injections were palatal, intraligamentary and intrapulpal injections, as required for each case. In the palatal infiltration technique, 0.5 ml of 3% prilocaine anaesthetic solution with felypressin 0.03 IU/mL was injected gently in the middle of the space between the midpalatal raphe and the palatal gingival margin using a 27-G, 25 mm length needle, while the needle tip was toward the bone. The needle penetration depth was based on preoperative periapical radiography. The tooth’s length was estimated using the parallel technique based on the periapical radiograph taken with an XCP (Dentsply Sirona, RINN, Konstanz, Germany). In maxillary first molar, since the malar bone process may prevent determining the needle penetration depth, the needle was penetrated as far as possible.

The needle for the maxillary second molar was penetrated based on the estimated working length. In case of anaesthesia failure and need for supplementary injection, the same syringe was used. The intraligamentary technique was performed with a 27-G, 25 mm length needle placed at an angle of 30 degrees to the longitudinal axis of the tooth, entering the mesial gingival sulcus with maximum penetration and 0.2 ml of 3% prilocaine anaesthetic solution with felypressin 0.03 IU/mL was injected with pressure. This was also done on the distal side of the tooth. The distinction between “success” and “failure” of supplementary injections was similar to the main infiltration technique. “Failures” of the main technique received supplementary injections, and “failures” of a supplementary technique received another injection. One week later, all patients were questioned to ask about any side effects from the infiltration and supplementary injections.

Continuous and categorical data were analysed with an independent t-test and a Chi-square test, respectively. P<0.05 was considered statistically significant. Statistical analysis was performed using SPSS software version 22 (IBM Corp., Armonk, NY, USA).

## Results

One hundred fifty-nine patients were eligible to participate in this study. The patients’ characteristics, such as gender and mean age, are summarised in Table 1. There was no significant difference in gender, mean age, and the number of the first and second maxillary molars (P>0.05).

**Table 1.** Characteristics of the patients

Teeth	Maxillary first molar Mean (±SD)	Maxillary second molar Mean (±SD)	P-value
Characteristic			
Age (years)	39.61 (±13.08)	41.53 (±10.93)	0.318
Gender			
Male	26	27	0.500
Female	58	48
N	84	75

N: Number

At the beginning of the study, a palatal injection was used as a supplementary technique. However, since one patient felt discomfort following an injection into the soft palate when her maxillary second molar was being treated, supplementary injections were subsequently limited to intraligamentary and intrapulpal injections. Other than that, no side effects were reported by the patients. Overall, 56.6% of the teeth were successfully anaesthetised, with no significant difference between the genders (P>0.05). Table 2 illustrates the success rate of buccal infiltration and intraligamentary and intrapulpal injection in teeth that did not anaesthetise after buccal infiltration. Higher anaesthesia was achieved in maxillary second molars (60%) compared to first molars (53.6%); however, there was no significant difference (P>0.05). Overall, 50% of the intraligamentary injections and 97.9% of the intrapulpal injections were successful. There was also no significant difference between maxillary first and second molars when intraligamentary and intrapulpal injections were used.

**Table 2.** Success rates of different techniques in maxillary first and second molars

Teeth	Maxillary first molar	Maxillary second molar	P-value
	Success n (%)	Failure n (%)		Success n (%)	Failure n (%)
Techniques					
Infiltration	45 (53.6)	39 (46.4)	45 (60)	30 (40)	0.05
Intraligamentary*	9 (47.3)	10 (52.7)	10 (52.6)	9 (47.4)	0.05
Intrapulpal*	28 (100)	0 (0.0)	19 (95)	1 (5)	0.05

*The supplementary techniques only employed when the infiltration injections had been failed

## Discussion

The results of this study confirmed the primary and secondary hypotheses that there was no significant difference between maxillary first and second molars in terms of anaesthesia success rates following infiltration injections and supplementary injections (P>0.05).

Previous studies have reported a range of 30 to 100% success rate for maxillary first molars with irreversible pulpitis by using either 2% lidocaine with different concentrations of adrenaline or 4% articaine with 1:100000 adrenaline when infiltration injections were used as the primary anaesthetic technique (11-15). In this study, after injection of 3% prilocaine with 0.03 IU/mL felypressin, the anaesthesia success rate for maxillary first molars was 53.6% which is within the range reported for other anaesthetic agents (11-15, 22).

A few studies have evaluated the anaesthesia success rate for maxillary molars (11-15, 22), but no study has compared the anaesthesia success between maxillary first and second molars. Anatomical differences such as cortical plate thickness, root divergence, and the presence of the maxillary sinus between the buccal and palatal roots might have the potential influence on the anaesthesia success rate in maxillary molars. For example, Kang et al. (17) 2015 showed that the mesiobuccal root of the second maxillary molar has the thickest mean horizontal distance to the buccal cortical plate in the posterior maxillary segment. Anatomical considerations need to be considered in clinical practice, hence the focus of this study (16-19).

Two separate investigations reported no significant difference in anaesthesia success rates of maxillary molars following buccal infiltrations compared with buccal and palatal infiltrations (22, 23). Therefore, in this study, only buccal infiltrations were used.

There are several possible side effects when prilocaine is used as an anaesthetic agent. Methemoglobinemia and paresthesia are two important side effects (8). Moreover, it should be emphasised that anaesthetic solutions with felypressin should not be used for pregnant women since it may induce methemoglobinemia and premature delivery due to uterine contractions (24). In this study, the patients reported none of these side effects, but the sample size was limited to only 159 patients. The only side effect was using a palatal injection as the supplementary technique for a maxillary second molar that anesthetised soft palate. For that reason, only intraligamental and intrapulpal injections were subsequently used as supplementary injections.

The intraligamental injection has been the most popular supplementary technique amongst dentists in the USA (25). The technique is easy to use and can be administered even after a dental dam has been placed. However, the results of the present study showed that only 50% of intraligamental injections as a “supplementary” technique were successful, with no significant difference between the maxillary first and second molars (P>0.05). Previous investigations have reported anaesthesia success rates for intraligamentary injections to range from 31.8% to 82% in mandibular molars (6, 26-28). However, in these studies, mandibular molars were the teeth that were anaesthetised. The anatomic location of the teeth may have a significant impact on the success rate of intraligamentary injection due to the difference in bone density and cortical bone thickness (20). However, this study had no significant difference between maxillary first and second molars.

The Heft-Parker visual analogue scale (VAS) (29) has been used in most investigations of anaesthesia success rates for teeth with irreversible pulpitis by asking the patients to grade their pain during endodontic treatment (27, 30-33). It has been reported that the pain threshold is similar among individuals, but their pain tolerance is significantly different (34). However, whether the VAS scores give researchers any indication of the patients’ pain threshold or their pain tolerance is not clear. This study used a categorised scale and whether the patients needed further anaesthesia to evaluate the patients’ pain and anaesthesia success. The success of the injection was assumed if the patients had no or mild pain during access cavity preparation and root canal instrumentation and no need for a supplementary injection. In addition to the categorised pain scale, the treating practitioner’s observations of any SEM were also used to signify the presence of pain during treatment. Studies mainly use the SEM evaluation for pain perception in children (21, 35). For that reason, in addition to just asking about the patients’ pain quality and evaluating their pain score as a reference to needing supplementary anaesthesia, the practitioner offered another anaesthesia injection. This method seems more accurate since even mild pain in a patient with low pain tolerance may mean that the patient needs a supplementary injection. Moreover, if a patient rejected the first offer of a supplementary injection but the SEM observations were positive following the treatment, the practitioner would know that the patient either had a fear of receiving another injection or low pain tolerance. The advantage of this method of evaluating anaesthesia success is that it replicates the clinical scenario encountered by dentists during endodontic treatment.

The intrapulpal injection has been considered as the most successful technique among the various supplementary injections, although the most important drawback of this technique is the significant injection pain which is very important when managing patients with low pain tolerance (20). However, one study has suggested that placing 20% benzocaine on the pulp exposure may significantly decrease pain during the intrapulpal injection (36).

It has been reported that premedication can influence the success rate of anaesthesia in mandibular posterior teeth (37). One of the exclusion criteria in most investigations evaluating anaesthesia success rate in patients who have used analgesics less than 12 hours before the treatment. Patients with mild pain are less likely to use analgesics before treatment, in contrast, it can be assumed that most patients with moderate to severe spontaneous pain may use premedication. Therefore, only the patients with mild spontaneous pain were included.

The present study’s results showed that maxillary first molars had an anaesthesia success rate of 53.6% and second molars had a 60% anaesthesia success rate. Several investigations have reported an anaesthesia success rate on maxillary molars; however, some did not mention if they included or excluded patients based on their pain severity, i.e., mild, moderate, severe (11-13). Another study only included patients with mild pain (14). Results of studies that included emergency patients, i.e., patients with vital pulp with spontaneous moderate to severe pain, showed that buccal infiltration had a 54% anaesthesia success rate in maxillary first molars. However, they used 2% lidocaine with 1:200000 epinephrine in their study (22).

## Conclusion

This study showed no significant difference between maxillary first and second molars with irreversible pulpitis following buccal infiltration injections of 3% prilocaine with 0.03 IU/mL felypressin. In addition, there was no significant difference in anaesthesia success rate between maxillary first and second molars when intraligamentary and intrapulpal injections were also used.

### Disclosures

**Conflict of interest:** The authors deny any conflict of interest.

**Ethics Committee Approval:** This study was approved by the ethics committee of the Kerman University of Medical Sciences (Date: 29/07/2019, Number: IR.KMU.REC.1398.236).

**Peer-review:** Externally peer-reviewed.

**Financial Disclosure:** This study did not receive any financial support.

**Authorship contributions:** Concept – M.P.; Design – M.P.; Supervision – M.P.; Funding - None; Materials - None; Data collection &/or processing – I.S.; Analysis and/or interpretation – N.N.; Literature search – I.S.; Writing – I.S.; Critical Review – P.A.

## References

[1] Abbott PV, Parirokh M (2018). Strategies for managing pain during endodontic treatment.. Aust Endod J.

[2] Abazarpoor R, Parirokh M, Nakhaee N, Abbott PV (2015). A comparison of different volumes of articaine for inferior alveolar nerve block for molar teeth with symptomatic irreversible pulpitis.. J Endod.

[3] Shahi S, Rahimi S, Yavari HR, Ghasemi N, Ahmadi F (2018). Success rate of 3 injection methods with articaine for mandibular first molars with symptomatic irreversible pulpitis: A CONSORT randomized double-blind clinical trial.. J Endod.

[4] Topçuoğlu HS, Arslan H, Topçuoğlu G, Demirbuga S (2019). The effect of cryotherapy application on the success rate of inferior alveolar nerve block in patients with symptomatic irreversible pulpitis.. J Endod.

[5] Zanjir M, Lighvan NL, Yarascavitch C, Beyene J, Shah PS, Azarpazhooh A (2019). Efficacy and safety of pulpal anesthesia strategies during endodontic treatment of permanent mandibular molars with symptomatic irreversible pulpitis: a systematic review and network meta-analysis.. J Endod.

[6] Aggarwal V, Singla M, Miglani S, Kohli S (2019). Efficacy of articaine versus lidocaine administered as supplementary intraligamentary injection after a failed inferior alveolar nerve block: a randomized double-blind study.. J Endod.

[7] Larocca de Geus J, Nogueira da Costa JK, Wambier LM, Maran BM, Loguercio AD, Reis A (2020). Different anesthetics on the efficacy of inferior alveolar nerve block in patients with irreversible pulpitis: A network systematic review and meta-analysis.. J Am Dent Assoc.

[8] Parirokh M, V Abbott P (2014). Various strategies for pain-free root canal treatment.. Iran Endod J.

[9] Gazal G (2019). Is prilocaine safe and potent enough for use in the oral surgery of medically compromised patients.. Saudi Med J.

[10] Sherman MG, Flax M, Namerow K, Murray PE (2008). Anesthetic efficacy of the Gow-Gates injection and maxillary infiltration with articaine and lidocaine for irreversible pulpitis.. J Endod.

[11] Srinivasan N, Kavitha M, Loganathan CS, Padmini G (2009). Comparison of anesthetic efficacy of 4% articaine and 2% lidocaine for maxillary buccal infiltration in patients with irreversible pulpitis.. Oral Surg Oral Med Oral Pathol Oral Radiol Endod.

[12] Kanaa MD, Whitworth JM, Meechan JG (2012). A comparison of the efficacy of 4% articaine with 1:100,000 epinephrine and 2% lidocaine with 1:80,000 epinephrine in achieving pulpal anesthesia in maxillary teeth with irreversible pulpitis.. J Endod.

[13] Atasoy Ulusoy Öİ, Alaçam T (2014). Efficacy of single buccal infiltrations for maxillary first molars in patients with irreversible pulpitis: a randomized controlled clinical trial.. Int Endod J.

[14] Hosseini HR, Parirokh M, Nakhaee N, V Abbott P, Samani S (2016). Efficacy of articaine and lidocaine for buccal infiltration of first maxillary molars with symptomatic irreversible pulpitis: a randomized double-blinded clinical trial.. Iran Endod J.

[15] Moradi Askari E, Parirokh M, Nakhaee N, Hosseini HR, Abbott PV (2016). The effect of maxillary first molar root length on the success rate of buccal infiltration anesthesia.. J Endod.

[16] Jung YH, Cho BH (2012). Assessment of the relationship between the maxillary molars and adjacent structures using cone beam computed tomography.. Imaging Sci Dent.

[17] Kang SH, Kim BS, Kim Y (2015). Proximity of posterior teeth to the maxillary sinus and buccal bone thickness: a biometric assessment using cone-beam computed tomography.. J Endod.

[18] Zhang X, Li Y, Zhang Y, Hu F, Xu B, Shi X (2019). Investigating the anatomical relationship between the maxillary molars and the sinus floor in a Chinese population using cone-beam computed tomography.. BMC Oral Health.

[19] Makris LML, Devito KL, D'Addazio PSS, Lima CO, Campos CN (2020). Relationship of maxillary posterior roots to the maxillary sinus and cortical bone: a cone beam computed tomographic study.. Gen Dent.

[20] Meechan JG (2002). Supplementary routes to local anaesthesia.. Int Endod J.

[21] Hameed NN, Sargod SS, Bhat SS, Hegde SK, Bava MM (2018). Effectiveness of precooling the injection site using tetrafluorethane on pain perception in children.. J Indian Soc Pedod Prev Dent.

[22] Aggarwal V, Singla M, Miglani S, Ansari I, Kohli S (2011). A prospective, randomized, single-blind comparative evaluation of anesthetic efficacy of posterior superior alveolar nerve blocks, buccal infiltrations, and buccal plus palatal infiltrations in patients with irreversible pulpitis.. J Endod.

[23] Guglielmo A, Drum M, Reader A, Nusstein J (2011). Anesthetic efficacy of a combination palatal and buccal infiltration of the maxillary first molar.. J Endod.

[24] Singh P (2012). An emphasis on the wide usage and important role of local anesthesia in dentistry: A strategic review.. Dent Res J (Isfahan).

[25] Bangerter C, Mines P, Sweet M (2009). The use of intraosseous anesthesia among endodontists: results of a questionnaire.. J Endod.

[26] Lin S, Wigler R, Huber R, Kaufman AY (2017). Anaesthetic efficacy of intraligamentary injection techniques on mandibular molars diagnosed with asymptomatic irreversible pulpitis: A retrospective study.. Aust Endod J.

[27] Aggarwal V, Singla M, Miglani S, Kohli S, Sharma V, Bhasin SS (2018). Does the volume of supplemental intraligamentary injections affect the anaesthetic success rate after a failed primary inferior alveolar nerve block? A randomized-double blind clinical trial.. Int Endod J.

[28] Aggarwal V, Singla M, Saatchi M, Hasija M (2020). Anaesthetic efficacy of 2% lidocaine with different concentrations of epinephrine (1:80,000 and 1:200,000) in intraligamentary injection after a failed primary inferior alveolar nerve block: a randomized double-blind study.. Acta Odontol Scand.

[29] Heft MW, Parker SR (1984). An experimental basis for revising the graphic rating scale for pain.. Pain.

[30] Parirokh M, Yosefi MH, Nakhaee N, Manochehrifar H, Abbott PV, Reza Forghani F (2012). Effect of bupivacaine on postoperative pain for inferior alveolar nerve block anesthesia after single-visit root canal treatment in teeth with irreversible pulpitis.. J Endod.

[31] Aggarwal V, Singla M, Miglani S, Kohli S (2014). Comparison of the anaesthetic efficacy of epinephrine concentrations (1 : 80 000 and 1 : 200 000) in 2% lidocaine for inferior alveolar nerve block in patients with symptomatic irreversible pulpitis: a randomized, double-blind clinical trial.. Int Endod J.

[32] Parirokh M, Sadr S, Nakhaee N, Abbott PV, Askarifard S (2014). Efficacy of supplementary buccal infiltrations and intraligamentary injections to inferior alveolar nerve blocks in mandibular first molars with asymptomatic irreversible pulpitis: a randomized controlled trial.. Int Endod J.

[33] Kumar U, Aggarwal V, Singh S, Singh SP, Gauba K (2020). Is bilateral mental incisive nerve block better than unilateral mental incisive nerve block during the endodontic management of mandibular incisors with symptomatic irreversible pulpitis? A prospective single-blind randomized clinical trial.. J Endod.

[34] Dawson A, List T (2009). Comparison of pain thresholds and pain tolerance levels between Middle Easterners and Swedes and between genders.. J Oral Rehabil.

[35] Aminabadi NA, Farahani RM (2009). The effect of pre-cooling the injection site on pediatric pain perception during the administration of local anesthesia.. J Contemp Dent Pract.

[36] Sooraparaju SG, Abarajithan M, Sathish ES, Suryakumari NB, Ealla KK, Gade W (2015). Anaesthetic efficacy of topical benzocaine gel combined with hyaluronidase for supplemental intrapulpal injection in teeth with irreversible pulpitis- a double blinded clinical trial.. J Clin Diagn Res.

[37] Parirokh M, Ashouri R, Rekabi AR, Nakhaee N, Pardakhti A, Askarifard S (2010). The effect of premedication with ibuprofen and indomethacin on the success of inferior alveolar nerve block for teeth with irreversible pulpitis.. J Endod.

